# Shared decision-making implementation status among dermatologists engaging in medical esthetics: a cross-sectional study in China

**DOI:** 10.3389/fmed.2024.1418917

**Published:** 2024-07-31

**Authors:** Shiyuan Li, Jing Fan, Yan Qiang, Zhen Duan, Ruiping Wang

**Affiliations:** ^1^Clinical Research Center, Shanghai Skin Diseases Hospital, School of Medicine, Tongji University, Shanghai, China; ^2^School of Public Health, Shanghai University of Traditional Chinese Medicine, Shanghai, China; ^3^Bloomberg School of Public Health, University of Johns Hopkins, Baltimore, MD, United States

**Keywords:** medical esthetics, cross-sectional study, good practice, implementation status, shared decision-making

## Abstract

**Objective:**

Shared decision-making (SDM) is a collaborative process in which patients and healthcare providers jointly make a medical decision. This cross-sectional study aimed to identify the implementation status of shared decision-making among dermatologists engaging in medical esthetics in China and to identify factors associated with the good practice of SDM among them.

**Methods:**

From January to June 2023, a total of 1,287 dermatologists engaging in medical esthetics in China were recruited and completed the online interviews about their implementation of SDM based on the Shared Decision-Making Questionnaire for Doctors (SDM-Q-Doc). Logistic regression was used to calculate the odds ratio (OR) and 95% confidence interval (CI) to explore factors associated with the higher SDM score achievement among dermatologists with medical esthetic practice.

**Results:**

The median value of the total SDM score was 39, and 48% (621/1278) of dermatologists with medical esthetic practice achieved at least 40 out of 45 scores. Logistic regression indicated that dermatologists aged 40–49 or ≥ 50 years and those engaging in medical esthetic practice for ≥5 years were more likely to achieve at least 40 out of 45 scores compared to dermatologists aged <30 years with less than 5 years of medical esthetic practice. The ORs were 1.82 (95% CI: 1.13–3.12), 1.94 (95% CI: 1.13–3.61), and 1.76 (95% CI: 1.34–2.31), respectively.

**Conclusion:**

The SDM implementation level among Chinese dermatologists engaging in medical esthetics is high, especially for those who are older age and have more years of practice. Hence, it is highly recommended to promote and enhance SDM practice among younger dermatologists engaging in medical esthetics with less working experience.

## 1 Introduction

In recent years, a growing number of patients are inclined to participate in the medical diagnosis and treatment process (1). The traditional paternalistic diagnosis and treatment relationship, led by doctors without sufficient patient–doctor information communication, can no longer adapt to the current medical market development (2). Therefore, shared decision-making (SDM) has been developed and integrated into clinical practice. SDM is a medical choice process based on the joint participation of doctors and patients, the exchange of information between them, and the consideration of various possible medical outcomes. In the decision-making process, doctors respect patients’ wishes and expect to get the most suitable medical plan for patients based on mutual understanding (3). There are four characteristics of SDM: (1) at least two participants–physician and patient be involved; (2) both parties share information; (3) both parties take steps to build a consensus about the preferred treatment; and (4) an agreement is reached on the treatment to implement (4).

In medical esthetics, which covers a full range of professional treatments to restore and maintain beauty, balance, and youthfulness, the application of SDM reflects the core value of decision-making because the views, opinions, and satisfaction among patients or beauty seekers play a very important role in the decision-making process of treatment options. Globally, medical esthetic practitioners have recognized the importance of SDM^4^ and applied SDM into clinical practice (5). SDM has been recommended in medical esthetic guidelines, including those for oncoplastic breast surgery (6). In the study of Alderman (7), patients who were well informed of reconstructive options were four times more likely to undergo mastectomy (with IBR) than oncoplastic breast-conserving surgery.

A survey among patients consulting esthetic surgery and their physicians in charge suggests that SDM helps improve patients’ satisfaction and ability to measure their own preferences and needs and helps physicians understand patients’ expectations and preferences (8). A scoping review of the role of shared decision-making in dental implant consultations emphasizes that shared decision-making has been shown to improve healthcare quality and increase clinician and patient satisfaction (9).

Although the medical esthetics is well suited for the implementation of SDM, SDM is not integrated into every physician’s clinical practice. A survey (10) among dermatologists found that potential hindrances to SDM implementation in medical esthetics were patients’ misconceptions, inadequate patient education materials, patient indecision, and limited communication time for physicians. Another survey (8) showed that postoperative appearance, surgical plan, and medical cost were the main influencing factors.

In China, SDM has limited practice in clinical circumstances (11). A study (12) suggested that there are several significant barriers to overcome before this aspiration becomes a reality. Doctor–patient relationships in China are relatively poor; consultations are often brief transactions. There is a huge market for medical esthetics in China. Different from ordinary patients, beauty seekers have a strong desire to participate in SDM, and SDM can reduce medical disputes and improve the level of satisfaction. However, there is limited research in this field in China. In this study, we aimed to understand the implementation of SDM in medical esthetics and to explore factors associated with the good practice of SDM in China.

## 2 Methods

### 2.1 Study population

**Figure 1:**
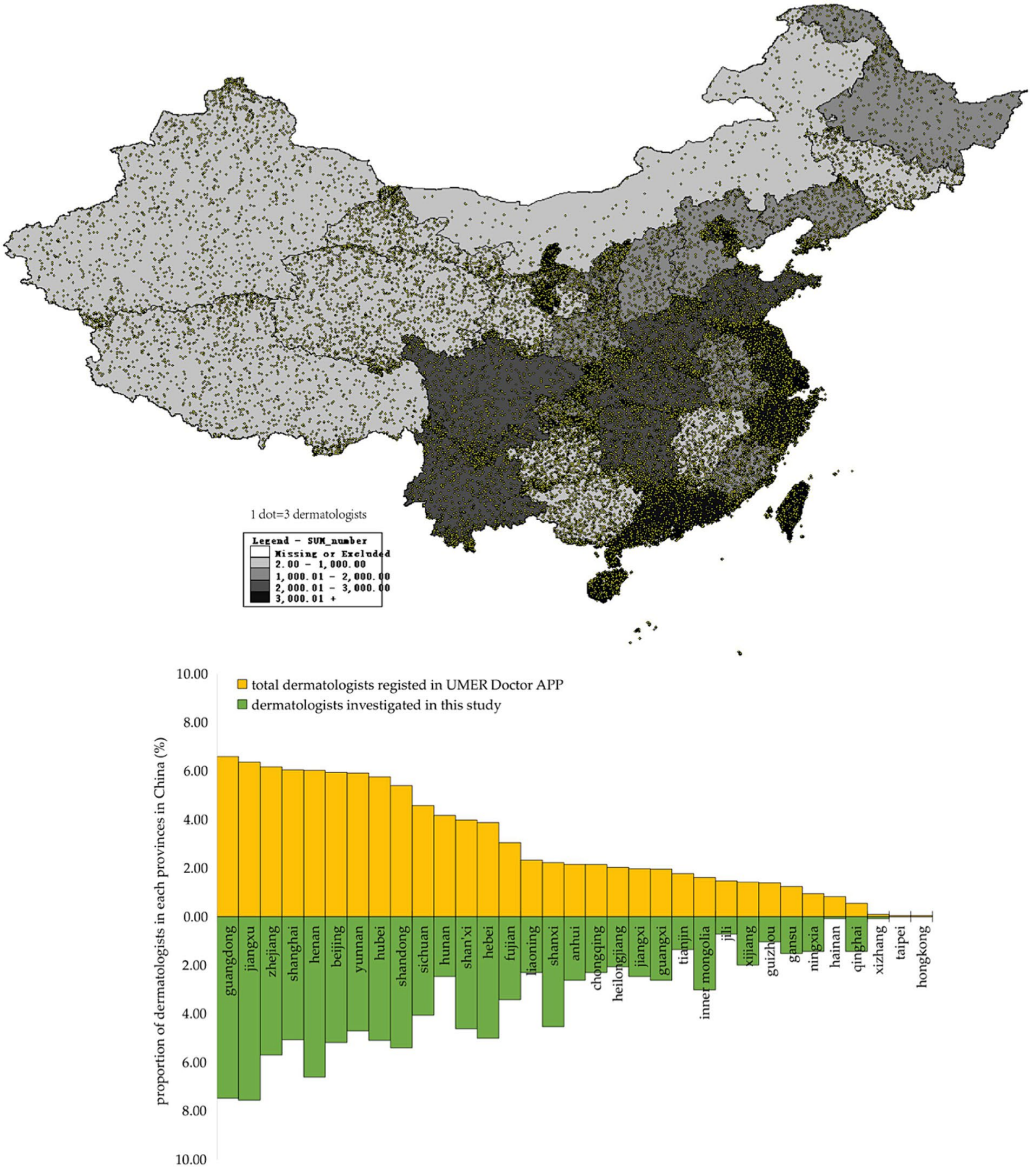
Regional distribution of dermatologists investigated in this study in China and comparison by the province in China of dermatologists investigated in this study and total dermatologists registered in UMER Doctor APP.

This study is a cross-sectional survey involving 33 provinces in China (Figure 1). Referring to previous studies, the prevalence of good SDM implementation in medical esthetics in China was 30%, we set the significance level α =0.05, the permissible error δ = 10%*p = 3%, with a 10% non-response rate, and the sample size *n* = [μ^2^_ɑ/2_ × p(1-p)]/δ^2^, indicating that at least 1,075 dermatologists engaging in medical esthetics should be enrolled.

### 2.2 Dermatologists with medical esthetic practice enrollment

The study was administered online using the Umer Doctor APP (Shanghai Maise Information Technology Co., Ltd.) from January to June 2023. According to the data provided by the Umer Doctor APP, there are over 30, 000 dermatologists registered at Umer Doctor APP, which covers 33 provinces in China, and approximately 5, 000 of them were dermatologists with medical esthetic practice for at least 12 months. To provide an up-to-date description of SDM practice among dermatologists practicing medical esthetics, the inclusion criterion was being a dermatologist with an active license to practice medical esthetics in China for at least 12 months. In this study, we sent invitations to 1,500 dermatologists who were randomly selected from among the 5,000 dermatologists with medical esthetic practice through the Umer Doctor APP. After eliminating incomplete questionnaires and those without responses, 1,287 out of 1,500 were successfully recruited with a responding rate of 86%. Figure 1 shows that the 1,287 selected dermatologists with medical esthetic practice are a good representative of the total 30,000 dermatologists registered at the Umer Doctor APP. Finally, all 1,287 dermatologists who provided informed consent were analyzed. This study was reviewed and approved by the Institutional Review Board of Shanghai Skin Disease Hospital (2022–31), and this study strictly adhered to the Declaration of Helsinki.

### 2.3 Data collection

In this study, the data were collected through the online questionnaire on the Umer Doctor APP, which covered two parts: (1) demographic features including sex, age, esthetic practice years, education, professional qualification, type of healthcare setting, and ownership of healthcare setting; (2) the Shared Decision-Making Questionnaire for Doctors (SDM-Q-Doc) in the field of medical esthetics.

The SDM-Q-Doc includes nine items: (1) I made clear to my patients that a decision needs to be made; (2) I wanted to know exactly from my patients how he/she wants to be involved in making the decision; (3) I told my patients that there are different options for treating his/her medical condition; (4) I precisely explained the advantages and disadvantages of the treatment options to my patients; (5) My patients understand all the information; (6) I asked my patients which treatment option he/she prefers; (7) My patients and I thoroughly weighed the different treatment options; (8) My patients and I selected a treatment option together; (9) My patients and I reached an agreement on how to proceed. A pilot study indicated that the split-half reliability coefficient of the questionnaire was 0.86, and the construct content validity coefficient was 0.73.

### 2.4 Definition and index calculation

In this study, age was classified as <30, 30–39, 40–49, or ≥ 50 years, and the esthetic practice years were classified as less than 5 years or greater than or equal to 5 years. Education level was classified as college and lower, graduate, master, or doctor/PhD/post-doctor. We classified professional qualifications as resident, attending physician, associated chief physician, and chief physician. The type of healthcare setting was classified as a general hospital, specialized hospital, ambulatory center, or clinic. Healthcare setting ownership was classified as a public or private institution.

The response to each of the nine items for SDM-Q-Doc is assessed using a 6-point Likert scale where 0 corresponds to completely disagree, 1 to strongly disagree, 2 to somewhat disagree, 3 to somewhat agree, 4 to strongly agree, and 5 to completely agree. The total SDM score is a sum of the scores for each question, which ranges from 0 to 45, with a higher score indicating a higher implementation level of SDM in medical esthetic practice. In this study, we define the good practice of SDM when dermatologists achieved at least 40 out of 45 scores based on the SDM-Q-Doc.

### 2.5 Data analysis

SPSS 25.0 was employed for data analysis in this study. Quantitative variables are presented as mean and standard deviation (SD) or median and interquartile range (IQR) as appropriate, and we applied Student’s *t*-test or Mann–Whitney *U*-tests to examine the differences between groups for quantitative variables. Qualitative variables were described as frequency and proportion (%), and a chi-squared test was used for statistical significance tests between groups. Logistic regression was applied to calculate the odds ratio (OR) and 95% confidence interval (95% CI) to explore factors associated with the good practice of SDM among dermatologists, and confounders adjusted in the logistic regression model were those variables with *p* < 0.05 identified through univariable analysis. Bar and box plots were produced to show the distribution of SDM scores, based on the SDM-Q-Doc among dermatologists engaged in medical esthetics, categorized by different sex, age, and years of medical esthetic practice. In this study, a difference with *p* < 0.05 (two-tailed) was regarded as statistically significant.

## 3 Results

In this study, 1,287 dermatologists included 395 (31%) men and 892 (69%) women. The mean age was 35 years, and the proportion of dermatologists aged <30, 30–39, 40–49, and ≥ 50 years was 21, 44, 23, and 10%, respectively. The median years of medical esthetic practice were 5 (IQR: 2.0–10.0) and 48% of dermatologists with less than 5 years of esthetic practice. In this study, 63 (5%) dermatologists had college-level education or lower, 602 (47%) had graduate-level education, 579 (45%) had a master’s degree, and 43 (3%) held a PhD or post-doctoral degree. For professional qualification, there were 471 (37%) residents, 513 (40%) attending physicians, 237 (18%) associate chief physicians, and 66 (5%) chief physicians. The majority of dermatologists were enrolled in the general hospital (79%), and 88% of them were from public institutions. In this study, female dermatologists were younger, with fewer years of medical esthetic practice, had a high proportion of achieving master’s degree and higher education, and were likely to practice medical esthetics in general hospitals (Table 1).

**Table 1:** Demographic feature for 1,287 dermatologists who practice medical esthetics in China.

Variables	Dermatologist (*n* = 1,287)	Male dermatologist (*n* = 395)	Female dermatologist (*n* = 892)	*z/χ^2^*	*P*
Age (years), (median, IQR)	35.0 (30.0–42.0)	39.0 (32.0–46.0)	34.0 (29.0–40.0)	7.99	0.00
Age (years), *n* (%)				63.24	0.00
< 30	283 (21.9)	50 (12.7)	233 (26.1)		
30–39	572 (44.4)	155 (39.2)	417 (46.8)		
40–49	305 (23.7)	129 (32.7)	176 (19.7)		
≥ 50	127 (9.9)	61 (15.4)	66 (7.4)		
Esthetic practice years, (median, IQR)	5.0 (2.0–10.0)	5.0 (3.0–10.0)	4.0 (2.0–9.0)	3.85	0.00
Esthetic practice years, *n* (%)				11.91	0.00
< 5 years	611 (47.5)	159 (40.3)	452 (50.7)		
≥ 5 years	676 (52.5)	236 (59.7)	440 (49.3)		
Education, *n* (%)				55.30	0.00
College and lower	63 (4.9)	35 (8.9)	28 (3.1)		
Graduate	602 (46.8)	221 (55.9)	381 (42.7)		
Master	579 (45.0)	122 (30.9)	457 (51.2)		
Doctor/PhD/Post-doctor	43 (3.3)	17 (4.3)	26 (2.9)		
Professional qualification, *n* (%)				18.76	0.00
Resident	471 (36.6)	118 (29.9)	353 (39.6)		
Attending physician	513 (39.9)	160 (40.5)	353 (39.6)		
Associated chief physician	237 (18.4)	97 (24.6)	140 (15.7)		
Chief physician	66 (5.1)	20 (5.1)	46 (5.2)		
Type of healthcare setting, *n* (%)				16.17	0.00
General hospital	1,018 (79.1)	296 (74.9)	722 (80.9)		
Specialized hospital	164 (12.7)	50 (12.7)	114 (12.8)		
Ambulatory center	57 (4.4)	23 (5.8)	34 (3.8)		
Clinics	48 (3.7)	26 (6.6)	22 (2.5)		
Ownership of healthcare setting, *n* (%)				12.98	0.00
Public institution	1,126 (87.5)	326 (82.5)	800 (89.7)		
Private institution	161 (12.5)	69 (17.5)	92 (10.3)		

SD, Standard deviation; IQR, Interquartile range.

### 3.1 Shared decision-making score among dermatologists based on SDM-Q-doc

**Figure 2:**
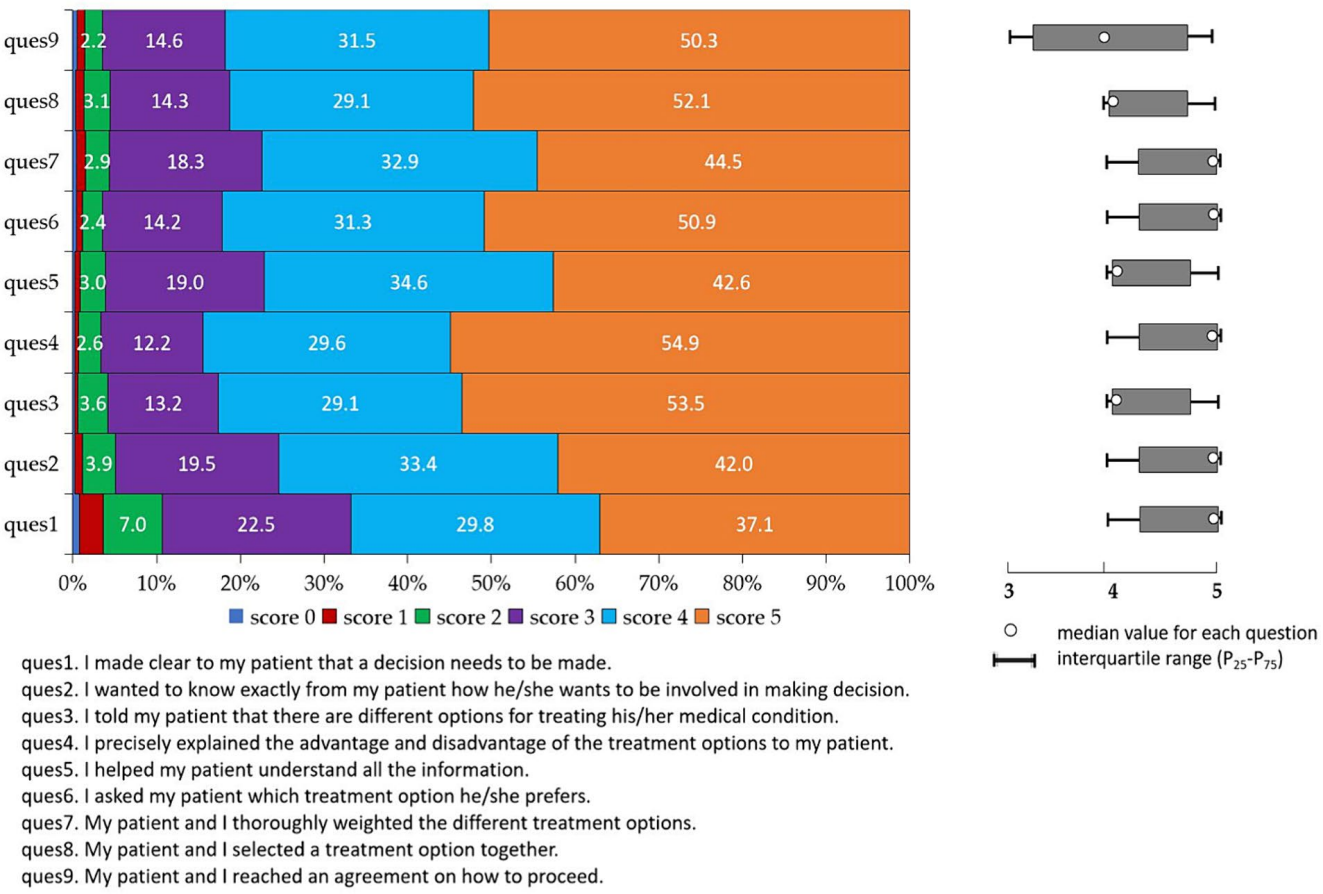
Score distribution of nine items of SDM-Q-Doc.

In this study, the median SDM score based on SDM-Q-Doc evaluation was 39 (IQR: 34–43). Data in Figure 2 show the detailed SDM score for the 9 items of SDM-Q-Doc, over 70% of dermatologists responded to each item of SDM with a score of 4 or 5, and the median score of the nine items of SDM ranged from 4 to 5. Data in Table 2 indicate that male dermatologists achieved a median SDM score of 39, which was similar to that among female dermatologists. Dermatologists who are older and have more years of esthetic practice achieved higher SDM scores, while dermatologists with a professional qualification of resident achieved the lowest SDM scores (*p* < 0.05; Table 2).

**Table 2:** Total shared decision-making (SDM) score based on SDM-Q-Doc among the 1,287 dermatologists who practice medical esthetics in China.

Variables	Total SDM scores median (P_25_-P_75_)	SDM Scores divided by quartile (P_25_, P_50_, P_75_), n (%)				*P1*	*P2*
		Less than 35	35–39	40–43	>43		
Sex						0.97	0.80
Male	39.0 (35.0–43.0)	96 (24.3)	109 (27.6)	96 (24.3)	94 (23.8)		
Female	39.0 (34.0–43.0)	235 (26.4)	226 (25.3)	218 (24.4)	213 (23.9)		
Age (years)						0.00	0.00
< 30	36.0 (32.0–42.0)	96 (33.9)	87 (30.7)	47 (16.6)	53 (18.7)		
30–39	39.0 (34.0–43.0)	155 (27.1)	143 (25.0)	144 (25.2)	130 (22.7)		
40–49	40.0 (36.0–44.0)	61 (20.0)	73 (23.9)	87 (28.5)	84 (27.5)		
≥50	42.0 (36.0–45.0)	19 (14.9)	32 (25.2)	36 (28.4)	40 (31.5)		
Esthetic practice years						0.00	0.00
< 5 years	37.0 (32.0–42.0)	209 (34.2)	166 (27.2)	125 (20.5)	111 (18.2)		
≥ 5 years	41.0 (36.0–44.0)	122 (18.1)	169 (25.0)	189 (27.9)	196 (29.0)		
Education						0.48	0.21
College and lower	40.0 (35.0–43.0)	15 (23.8)	13 (20.6)	20 (31.8)	15 (23.8)		
Graduate	39.0 (35.0–44.0)	147 (24.4)	155 (25.8)	143 (23.8)	157 (26.1)		
Master	39.0 (34.0–43.0)	155 (26.8)	155 (26.8)	141 (24.4)	128 (22.1)		
Doctor/PhD/Post-doctor	39.0 (31.0–43.0)	14 (32.6)	12 (27.9)	10 (23.3)	7 (16.3)		
Professional qualification						0.00	0.00
Resident	37.0 (32.0–43.0)	155 (32.9)	126 (26.8)	94 (20.0)	96 (20.4)		
Attending physician	40.0 (35.0–44.0)	120 (23.4)	129 (25.2)	134 (26.1)	130 (25.3)		
Associated chief physician	40.0 (36.0–44.0)	48 (20.3)	64 (27.0)	64 (27.0)	61 (25.7)		
Chief physician	42.0 (36.0–44.0)	8 (12.1)	16 (24.2)	22 (33.3)	20 (30.3)		
Type of healthcare setting						0.86	0.69
General hospital	39.0 (34.0–43.0)	270 (26.5)	261 (25.6)	246 (24.2)	241 (23.7)		
Specialized hospital	39.0 (36.0–43.0)	31 (18.9)	55 (33.5)	47 (28.7)	31 (18.9)		
Ambulatory center	40.0 (32.0–44.0)	18 (31.6)	9 (15.8)	11 (19.3)	19 (33.3)		
Clinics	40.0 (34.0–45.0)	12 (25.0)	10 (20.8)	10 (20.8)	16 (33.3)		
Healthcare setting ownership						0.78	0.89
Public institution	39.0 (34.0–43.0)	290 (25.8)	294 (26.1)	271 (24.1)	271 (24.1)		
Private institution	39.0 (34.0–43.0)	41 (25.5)	41 (25.5)	43 (26.7)	36 (22.4)		

SD, Standard deviation; IQR, Interquartile range; SDM, shared decision-making; P1, *p*-value based on Kruskal–Wallis test; P2, *p*-value based on Cochran–Mantel–Haenszel test (chi-square test).

**Figure 3:**
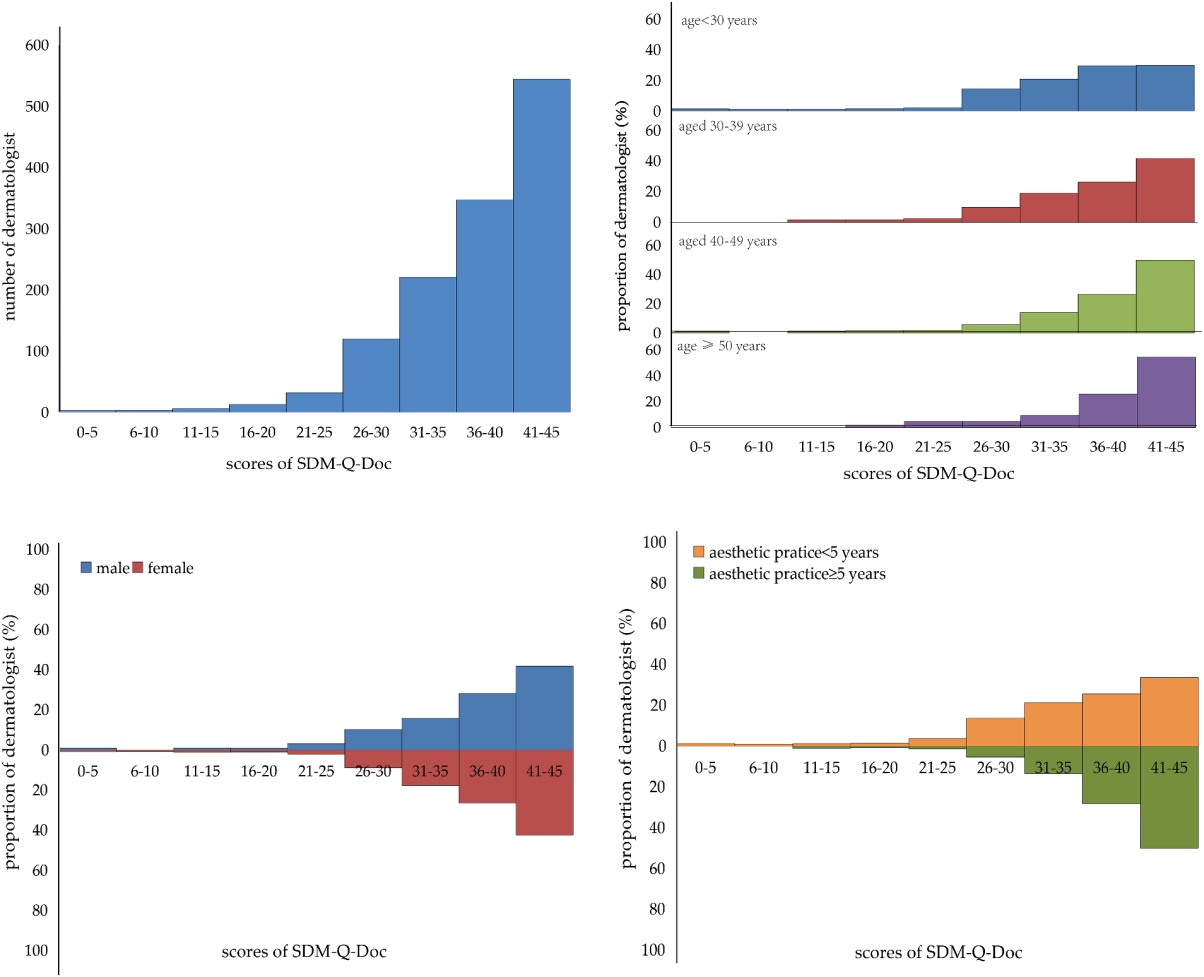
Distribution of the total score of SDM-Q-Doc and the distribution of the total score of SDM-Q-Doc in different age groups, different sex groups, and different esthetic practice year’s groups.

In this study, 621 dermatologists achieved at least 40 out of 45 scores based on SDM-Q-Doc evaluation, and the proportion of good SDM practice was 48% (621/1278). Among the 1,278 dermatologists practicing medical esthetics, the proportion of good SDM practice was similar between male (48.1%) and female (48.3%) dermatologists. The proportion of good SDM practice among dermatologists aged <30, 30–39, 40–49, and ≥ 50 was 36, 48, 56 and 60%, respectively. Dermatologists with ≥5 years of esthetic practice had a higher proportion of good SDM practice (57%) than those with <5 years of esthetic practice. For dermatologists with different professional qualifications, residents had a lower prevalence of good SDM practice (40%) than attending physicians (52%), associated chief physicians (53%), and chief physicians (64%). However, the proportion of good SDM practice among dermatologists with different education, different types of healthcare settings, and different healthcare setting ownership were not statistically significant (*p* < 0.05; Table 3; Figure 3).

**Table 3:** Proportion of dermatologists who achieved 40 out of 45 scores based on SDM-Q-Doc evaluation and its associated influencing factors among those who practice medical esthetics in China.

Variables	Proportion of dermatologists with 40 out of 45 scores		Model 1		Model 2		Model 3	
	Number	%	OR	95% CI	OR	95% CI	OR	95% CI
Sex								
Male	190	48.1	1.00		1.00		1.00	
Female	431	48.3	1.01	0.80–1.28	1.16	0.89–1.49	1.17	0.91–1.50
Age (years)‡								
< 30	100	35.3	1.00		1.00		1.00	
30–39	274	47.9	***1.68***	***1.25–2.26***	1.38	0.93–2.04	1.34	0.92–1.96
40–49	171	56.1	***2.24***	***1.68–3.26***	***1.87***	***1.13–3.12***	***1.82***	***1.13–2.94***
≥ 50	76	59.8	***2.73***	***1.77–4.19***	***1.99***	***1.01–3.93***	***1.94***	***1.04–3.61***
Esthetic practice years‡								
< 5 years	236	38.6	1.00		1.00		1.00	
≥ 5 years	385	57.0	***2.10***	***1.68–2.63***	***1.78***	***1.35–2.35***	***1.76***	***1.34–2.31***
Professional qualification‡								
Resident	190	40.3	1.00		1.00		1.00	
Attending physician	264	51.5	***1.57***	***1.22–2.02***	0.98	0.69–1.39	0.99	0.71–1.38
Associated chief physician	125	52.7	***1.65***	***1.21–2.26***	0.79	0.49–1.27	0.78	0.50–1.22
Chief physician	42	63.6	***2.59***	***1.52–4.42***	1.12	0.54–2.30	1.10	0.56–2.18
Education								
College and lower	35	55.6	1.00		1.00			
Graduate	300	49.8	0.80	0.47–1.34	0.80	0.45–1.43		
Master	269	46.5	0.69	0.41–1.17	0.89	0.48–1.66		
Doctor/PhD/Post-doctor	17	39.5	0.52	0.24–1.15	0.61	0.26–1.45		
Type of healthcare setting								
General hospital	487	47.8	1.00		1.00			
Specialized hospital	78	47.6	0.99	0.71–1.38	0.93	0.66–1.31		
Ambulatory center	30	52.6	1.21	0.71–2.07	1.13	0.63–2.02		
Clinics	26	54.2	1.29	0.72–2.30	1.20	0.62–2.36		
Healthcare setting ownership								
Public institution	542	48.1	1.00		1.00			
Private institution	79	49.1	1.04	0.75–1.44	0.84	0.56–1.27		

‡The difference between groups was statistically significant. OR, Odds ratio; CI, Confidence interval; SDM, shared decision-making; Model 1: Univariate logistic regression for factors associated with dermatologists with higher SDM scores (40/45). Model 2: Multivariate logistic regression with all variables incorporated into the model (sex, age, esthetic practice year, professional qualification, education, type of healthcare setting, and ownership of healthcare setting). Model 3: Multivariate logistic regression with the adjustment of sex, age, esthetic practice year, and professional qualification. Bold values means the difference between groups was statistically significant.

### 3.2 Factors associated with good SDM practice among dermatologists practicing medical esthetics

The univariate logistic regression results are depicted in model 1 in Table 3. Dermatologists aged 30–39, 40–49, or ≥ 50 years were more likely to have a higher proportion of good SDM practice than dermatologists aged <30 years, the ORs were 1.68 (95% CI: 1.25–2.26), 2.24 (95% CI: 1.68–3.26) and 2.73 (95% CI: 1.77–4.19), respectively. Dermatologists engaging in esthetic practice for ≥5 years had a higher proportion of achieving good SDM practice (OR = 2.1; 95% CI: 1.68–2.63) than those with <5 years. Moreover, compared to dermatologists with resident professional qualifications, dermatologists with attending physician, associated chief physician, or chief physician professional qualifications were more likely to have a higher proportion of good SDM practice; the OR was 1.57 (95% CI: 1.22–2.02), 1.65 (95% CI: 1.21–2.26), and 2.59 (95% CI: 1.52–4.42), respectively (Table 3).

Model 2 in Table 3, dermatologists aged 40–49 or ≥ 50 years were more likely to have a higher proportion of good SDM practice than dermatologists aged <30 years; the ORs were 1.87 (95% CI: 1.13–3.12), and 1.99 (95% CI: 1.01–3.93), respectively. Dermatologists with ≥5 years of esthetic practice had a higher proportion of achieving good SDM practice (OR = 1.78; 95% CI: 1.35–2.35) than those with <5 years of esthetic practice.

Model 3 in Table 3 was the logistic regression analysis results with the adjustment of age, sex, esthetic practice year, and professional qualification. Dermatologists aged 40–49 or ≥ 50 years were more likely to have a higher proportion of good SDM practice than dermatologists aged <30 years; the ORs were 1.82 (95% CI: 1.13–2.94) and 1.94 (95% CI: 1.04–3.61), respectively. Moreover, dermatologists with ≥5 years of medical esthetic practice had a higher proportion of achieving good SDM practice (OR = 1.76; 95% CI: 1.34–2.31) than those with <5 years of esthetic practice (Table 3).

## 4 Discussion

To our knowledge, this is the first cross-sectional study that recruited 1,287 dermatologists engaging in medical esthetics to explore the implementation status of shared decision-making in medical esthetics in China. The findings suggest that the SDM implementation among Chinese dermatologists engaged in medical esthetics is high, especially among those who are older and have more years of medical esthetic practice.

In this study, the median value of the total SDM score was 39 based on SDM-Q-Doc evaluation, which accounted for 87% of the total 45 scores. In an exploratory study in anesthesiology (13), the median SDM score among surgeons was 84% of the total 45 score, which was also high and in line with the findings of our study. However, a study (14) of the SDM in pediatric otolaryngology surgical consultations showed a relatively lower SDM score (70%), which might be due to the neglect of pediatric patients’ opinions by physicians. This study focused on medical esthetics, beauty seekers are always more proactive about the treatment, and their enthusiasm might affect doctors’ SDM implementation level. The observed levels of SDM increased with consultation length (14). Although this study indicated that the SDM implementation level among Chinese dermatologists with medical esthetic practice is high, it is important to note that doctors may tend to overestimate themselves and give themselves higher scores than what is accurate. Therefore, the SDM score based solely on the SDM-Q-Doc evaluation may not fully reflect the actual status of SDM implementation in the medical esthetics industry in China (15). That is, although dermatologists tend to be satisfied with their SDM, they are unlikely to be fully effective in communicating different treatment options to patients, and patients do not necessarily perceive this to be the case. Hence, there is still room for improvement in the following aspects, such as the clarity of explaining decisions to patients, the willingness of dermatologists to accept patients’ decisions, the comprehensiveness of dermatologists’ efforts to ensure patients understand the decision-making information, and the process of reaching the final decision jointly.

In this study, we noticed that the SDM implementation level among Chinese dermatologists who are older and have more years of esthetic practice is high, and they tended to have a higher prevalence of good practice of SDM in medical esthetics. Young HN (16) reported that the age of the doctor was a significant factor influencing SDM implementation, with older doctors tending to implement SDM at a higher level in medical esthetic practice. Fukui (17) implemented a study to explore the predictors of SDM and the level of agreement between consumers and providers in psychiatric care, they also noticed that the age and work experience of SDM providers were associated with the level of SDM implementation, although without statistical significance. This might be because practitioners who are older and have more years of practice tended to understand all aspects of medical esthetic decision-making, their ability to explain decisions has been enhanced, and their understanding of doctor–patient relationship and their emphasis on SDM has also been enhanced. With the enhancement of practitioners’ discourse power in medical esthetics, their preparation time for SDM has also been increased, all of which contributed to the higher SDM score (18, 19). Given this phenomenon, it is necessary to strengthen the education and training of young dermatologists on SDM, to enhance their attention to SDM, and to improve the level of SDM in China’s medical esthetic industry.

This study has some limitations. First, data were collected through online investigation, which may weaken the representative of this study and induce information bias even with a response rate of 86% among the 1,500 randomly invited dermatologists. Dermatologists with medical esthetic practice who are more positive to SDM may be prone to respond to this survey, while those who do not know or are negative to SDM may not respond. Second, this study missed the patient component, previous studies showed that the involvement of patients’ interaction provides a holistic understanding of SDM practice. Given the sample size is over 1,000 in this study, it is difficult to enroll the same number of patients to explore the interaction, which is an inevitable challenge. Third, this study had information bias such as over-reporting, and we measured the SDM implementation through self-reported questionnaires by the SDM-Q-DOC scale, which may raise the possibility of respondents over-reporting their SDM implementation. Therefore, the incorporation of some improvements such as face-to-face interviews and patient components in future studies could ensure an in-depth understanding of SDM practice among dermatologists with medical esthetic practice in China.

## 5 Conclusion

The SDM implementation level among Chinese dermatologists engaging in medical esthetics is high, especially for those who are older and have more years of practice. In clinical practice, dermatologists should emphasize clarity in explaining decisions to patients, the willingness of dermatologists to accept patients’ decisions, and the final decision reached together. Moreover, it is highly recommended to promote and enhance SDM practice among younger dermatologists with less working experience.

## Data availability statement

The raw data supporting the conclusions of this article will be made available by the authors, without undue reservation.

## Ethics statement

The study was reviewed and approved by the Review Board of Shanghai Skin Diseases Hospital of Tongji University (2022–31). Informed consent was signed online by each participant before the questionnaire interview. The studies were conducted in accordance with the local legislation and institutional requirements. The participants provided their written informed consent to participate in this study. Written informed consent was obtained from the individual(s) for the publication of any potentially identifiable images or data included in this article.

## Author contributions

SL: Writing – original draft, Formal analysis, Data curation. JF: Writing – review & editing, Project administration. YQ: Writing – review & editing, Supervision, Data curation. ZD: Writing – review & editing, Data curation. RW: Writing – review & editing, Supervision, Project administration, Data curation, Conceptualization.
